# Maximising translational value of the Iowa gambling task in preclinical studies through the use of the rodent touchscreen

**DOI:** 10.3389/fpsyt.2025.1518435

**Published:** 2025-01-27

**Authors:** Judith A. Pratt, Brian J. Morris

**Affiliations:** ^1^ Strathclyde Institute of Pharmacy and Biomedical Sciences, University of Strathclyde, Glasgow, United Kingdom; ^2^ School of Psychology and Neuroscience, College of Medical, Veterinary and Life Sciences, University of Glasgow, Glasgow, United Kingdom

**Keywords:** gambling, touchscreen, dopamine, impulsivity, risk-reward

## Abstract

The Iowa gambling task is widely employed to assess the evaluation of risk versus reward contingencies, and how the evaluations are implemented to gain advantageous returns. The cognitive processes involved can be compromised in psychiatric conditions, leading to the development of analogous tasks with translational value for use in rodents. The rodent touchscreen apparatus maximises the degree of similarity with the human task, and in this review we provide an outline of the use of rodent touchscreen gambling tasks in preclinical studies of psychiatric conditions. In particular, we describe how the basic task has been adapted to probe the relative contributions of different neurotransmitter systems, and specific aspects of cognition. We then offer a perspective on how the task might be employed most beneficially in future studies.

## Introduction

The ability to make sound decisions is critical in everyday life and is influenced by cognitive control and emotional systems, underpinned by shifting influences of cortico-striato-thalamo-cortical networks. During decision-making, individuals evaluate the probabilities and risks associated with different options, and this includes during recreational pastimes such as gambling. Whilst gambling is often a harmless pursuit, for some decision making can become maladaptive, leading to a diagnosis of Gambling disorder, which is identified as a behavioural addiction under DSM-5 (1).

The Iowa gambling task (IGT) developed ~30 years ago by Bechara and co-workers (2), captures ‘real world’ decision making performance under ambiguous and risky conditions in a clinical laboratory setting. The IGT involves probabilistic learning via monetary reward and punishment. Participants choose from 4 decks of cards. Unbeknown to them, some decks are advantageous in the short term (large wins) but disadvantageous in the long term (large frequent losses) whereas others are less attractive in the short term (small wins) but advantageous over time (small, less frequent losses). This induces a conflict between immediate high rewards and long-term gains. Hence, the optimal strategy to maximise earnings on the task is to select cards that yield smaller gains over time but lower penalties and avoid the ‘high risk, high reward’ decks.

The dynamics of decision making in the IGT are complex and involve a range of neurophysiological processes, each underpinned by distinct neural networks. Participants must learn and remember the different contingencies as the task progresses, integrate affective and cognitive information over time into future strategy, along with inhibiting attractive but risky choices. Early ‘exploratory’ stages of the task that involve decision-making under uncertainty likely recruit the ‘emotional system’ and as the task progresses the ‘cognitive control’ system orchestrates instrumental behaviour to enable the best long-term option (3). As elegantly summarised throughout this Special Edition, the IGT has proved an important tool for probing aspects of cognitive function related to the perception and experience of risk and reward.

## Rodent models of the IGT

Rodent models of decision-making processes akin to the IGT are important in translational research, as these have the potential to increase understanding of the neurobiological mechanisms involved, which in turn can inform new treatments. The validity of animal models is frequently assessed against criteria of face, predictive and construct validity (4). Face validity is the similarity of what is observed in the animal model compared to human symptoms, predictive validity relates to the model’s potential to predict human processes, often with respect to identifying drug treatments, and construct validity signifies the extent to which the model has similar neurobiological processes to those in humans.

Several rodent versions of the IGT (rGT) have evolved, with an initial focus on maze-based non operant tasks and automated operant tasks in adapted 5-hole operant chambers (for reviews see (3, 5, 6). More recently, automated touchscreen operant tasks of the rGT have been adopted (7–11). In terms of satisfying criteria for animal models, these models exhibit good face validity despite differences in task features, training, single and multiple session learning processes and end point assessments (**Table 1**). Hence animals can make decisions in the face of uncertainty (similar to the human IGT) in the sense that they are able to evaluate which options are advantageous in the long-term and adapt their behaviour by avoiding risky options typically associated with larger rewards. All studies report that rats and mice successfully learn the task and show clear preference for the most optimal option. However, assessment of construct and predictive validity is more difficult to assess in these models, given the incomplete knowledge of the neurobiological processes in decision making and the lack of pharmacological treatments available for gambling. Features of construct validity will be discussed when relevant in evaluation of the tasks below. Nonetheless, the RDoC and CNTRICS initiatives have confirmed that the rGT has an important role to play in cross species translation (from rodent to human) of decision-making processes (12).

**Table 1:** Comparison of the characteristics of maze-based and operant rodent gambling tasks (5-hole chamber and Touchscreen) with the human IGT.

Ref	Equipment	Species	No of Choices	Reward	Punishment	Reward occurrence	Task Duration	Features of Task
Bechara et al., 1994 (2)	Card game (now computerised)	Human	4	Money gain	Money loss	Each Trial	Single session (100 trials)	Decision making under uncertainty; conflict between immediate gratification and long-term gains. Card choices from 4 decks of cards which result in winning or losing hypothetical money. Unbeknown to subjects, two card decks are ‘risky’ (large wins but larger losses) and two decks are ‘safe’ (gradual accumulation of wins and negligible losses). Magnitude and frequency of losses varies between the 2 “risky” and between the 2 “safe” decks
Van den Bos et al., 2006 (13)	Manually operated 8 arm maze	Rat and mouse	4 arms	Sucrose (sweet) pellets	Quinine (bitter) pellets	Alternating with punishment	12 daily sessions (10-20 trials)	Conflict between probability of high reward vs punishment (quinine). Uncertainty represented by varying sequence/position of sugar and quinine presentation between blocks of trials
Pittaras et al., 2020 (15)	4 arm maze	Mouse	4 arms	Food pellets	Quinine pellets	Each trial (immediate reward) followed by delayed reward (palatable or quinine)	Habituation in an operant chamber followed by 5 daily test sessions in maze (20 trials/day)	Similar protocol to van den Bos 2006. Choice between two disadvantageous (quinine coated pellets) arms and two advantageous (palatable pellets) arms.
Cabeza et al., 2020 (16)	4 arm maze	Mouse	4 arms	Food pellets	Quinine pellets	Each trial (immediate reward) followed by delayed reward (palatable or quinine)	Habituation in maze followed by 5 daily test sessions in maze (20 trials/day)	Choice between two disadvantageous arms and two advantageous arms, varying proportions of palatable and quinine-treated pellets).
Pais-Veira et al., 2007 (20)	2 lever operant chamber	Rat	2 chambers -each with lever	Sucrose pellets	No reward	Alternating with punishment	Training days followed by single probe session of 90 trials	Assess preference for infrequent large amount of food reward compared to more frequent, smaller amount of reinforcer. Similar amounts of total reward over the trial period. Assess shift in preference for reward contingencies associated with levers over single probe trial.
Rivalan et al., 2009 (18)	Automated Five hole operant chamber	Rat	4 Holes (central hole blocked) Nose pocks into holes.	Food pellets	Time-outs 6-12s or 222-444s	Each trial	Training days followed by single test session of 1 hr	Deduce by trial and error among 4 options the 2 that are most advantageous in the long term. 2 options – bigger immediate reward with higher unpredictable penalties (time-out) and 2 options with smaller rewards but shorter unpredictable penalties
Zeeb et al., 2009 (17)	Automated Five hole operant chamber	Rat	4 Holes (central hole blocked) Nose pocks into holes.	Sucrose pellets	Time-outs 5-40s	According to schedule	Training sessions followed by ~25 daily sessions (100 trials/30min)	As with Rivalon et al., 2009 but with differences in duration of training and reinforcement contingencies associated with the 4 options. Probability of receiving reward or punishment for each option remains constant throughout session. Multiple test sessions enable assessments of pharmacological agents/other manipulations.
Young et al., 2011 (21)	Automated five hole operant chamber	Mouse	4 nose poke holes (central hole blocked)	Strawberry milkshake	Time-outs	According to schedule	Training sessions to stable performance ~25 days	Schedule based upon Zeeb et al., 2009 for rats. Assessment of performance in GM mice.
Silveira et al., 2016 (61)	Automated five hole operant chamber	Rat	4 nose poke holes (central hole not used)	Sucrose pellets	Time-outs 5-40s	According to schedule	Training sessions to stable performance ~25 days	Schedule based upon Zeeb et al., 2009 for rats. Assessment of pharmacological agents
Kim et al., 2017 (11)	Automated touchscreen operant chamber	Rat	4 response windows.	Sucrose pellets	Time-outs 5-40s	According to schedule	Training sessions followed by 15 daily test sessions (~30min) to assess a rat’s risk preference -then drug challenge test (30min)	Schedule based upon Zeeb et al., 2009 Investigation of trait and housing conditions
Humby et al., 2020 (7)	Automated touchscreen operant chamber	Mouse	2	Condensed milk	Time-outs	According to schedule	Training followed by ‘no choice’ trials (to maintain responding) or choice trials (quit or gamble-associated visual stimulus). Multiple test sessions	Schedule incorporates loss-chasing options over repeated programmed losses. Enables loss chasing to be compared to initial decision to quit or gamble. Assessment of pharmacological agents
Elsilä et al., 2020 (8)	Automated touchscreen operant chamber	Mouse	4 response windows	Sucrose solution	Time-outs 5-40s	According to schedule	Training sessions followed by multiple test sessions (~20)	Contingencies based upon Zeeb et al., 2009. Four options that differ in magnitude of reward or punishment possibilities. Worst option – total 2400s time-out. Assessment of pharmacological agents
Thomson et al., 2021 (9)	Automated touchscreen operant chamber	Mouse	4 response windows	Strawberry milk shake	Time-outs	According to schedule	Training sessions followed by multiple test sessions (~20)	Contingencies based upon Zeeb et al., 2009. Four options that differ in magnitude of reward or punishment possibilities. Worst option – total 720s time-out. Assessment in GM mice.
Beyer et al., 2021 (64)	Automated touchscreen operant chamber	Rat	4 response windows	Sucrose pellets	Time-outs	According to schedule	Training sessions followed by multiple test sessions (~20)	Contingencies based upon Zeeb et al., 2009. Four options that differ in magnitude of reward or punishment possibilities. Worst option – total 444s time-out. Assessment in rats overexpressing D1 receptor
Openshaw et al., 2022 (10)	Automated touchscreen operant chamber	Mouse	4 response windows	Strawberry milk shake	Time-outs	According to schedule	Training sessions followed by multiple test sessions (>60)	Adaption of Thomson et al., 2021 to incorporate contingency -shifting option. Worst option – total 720s time-out. Assessment of phenotype and pharmacological agents in GM mice

Note in rodent tasks, animals require varying amount of pre-training and typically require food restriction (80-95% of free feeding weight to ensure animals are motivated to perform the task.

The first rGT protocol to be established was a non-automated 4 arm maze-based task for rats and mice (13, 14). The task measures choices between two goal arms for obtaining differing amounts of sugar pellet reward or punishment (bitter-tasting quinine pellet). For choices in the ‘advantageous’ arm there are low immediate rewards but with a net gain over time, whereas for choices in the ‘disadvantageous’ arm the chance of high immediate rewards is offset by high net loss in the long-term. Hence this task shows good face-validity as there is conflict between short-term and long-term pay off of choices as in the human IGT. Interestingly, there was a similar sex difference in reward-related decision making in this rat task as has been shown in humans (13). By comparison with operant based models and the human IGT, this paradigm does not differentiate between long-term outcome and frequency of options of reward/punishment. Variations of this non-operant maze-based task have been developed in mice (15, 16) with suggestions that the inter-individual differences in decision making offer good face-validity.

With the introduction of operant based automated tasks, there is the opportunity to investigate a wide range of neurocognitive parameters concurrently that are relevant to human gambling such as impulsivity, compulsivity and cognitive inflexibility The operant chamber rGT model introduced by Zeeb et al. (17) utilises the standard five-hole operant chamber typically used for the five-choice serial reaction time task adapted such that only four holes are used in the task. Since features of this task protocol have been adopted in recent touchscreen tasks for rats and mice, a brief resume of the face, predictive and construct validity of this task is described here. Animals have a choice between four distinct options which are similar to the four decks of cards in the IGT (face validity). The options differ in frequency and magnitude of reward/punishment possibilities. Nose poking in the two options which result in small numbers of sucrose pellets as rewards and short unpredictable penalties are ultimately advantageous compared to nose poking in the other two holes which result in higher numbers of rewards but higher unpredictable penalties (disadvantageous options). In this task, penalties are ‘time-out’ periods during which time no reward can be obtained and have been likened to ‘loss’ in the human task (18).

In this operant task, animals are first trained to make a basic operant response (nose-poke response) into an illuminated hole within a short timeframe, in order to receive a reward. Animals are then trained to experience four reinforcement contingencies in forced choice sessions before undertaking the decision-making tests. The advantage of this approach is that it ensures that all animals have equal exposure to the four different reinforcement contingencies and minimises any biases due to inadequate sampling. Thereafter the impact of manipulations (e.g. drugs or lesions) can be evaluated robustly using a “within subjects” design. Whilst it could be argued that task performance may relate more to the later phase of the IGT when contingencies are known (exploitation), the finding that choice options vary between sessions earlier in training suggests that exploration learning does occur. A further advantage of this rGT task is that, in addition to decision-making processes related to ‘risk/reward’ evaluation, output measures, including gambling-related premature responses (inhibitory/impulse control), perseverative (compulsive) behaviours, and also executive function (e.g. attention) can also be measured, thereby increasing face validity.

Construct validity relates in part to similar neurobiological systems underpinning the behaviour in animal and human tasks. As with human studies, components of the cortico-striato-thalamo-cortical circuits have been identified in the rGT (5). For example, lesion studies in rats (19, 20) have shown the amygdala and orbitofrontal cortex (OFC) to differentially affect exploration and exploitation phases of the operant based rat gambling task.

The development of mouse paradigms is important for improving construct validity since they allow the use of genetic manipulations (GM), optogenetic and imaging tools to probe neural systems, genetic and environmental risk factors relevant to gambling disorders. Young et al. (21) utilised a mouse version of the validated operant task (17), to demonstrate that knockdown of the dopamine transporter in GM mice resulted in increased risk-taking behaviour. By comparison alpha-synuclein deletion mice showed decreased impulsive action (premature responding) without an effect on risky decision making (22). However, there has been limited use of this mouse operant paradigm to dissect the neural circuits and systems that underpin behaviours relevant to gambling.

## Touchscreen tasks – translational capacity

There is a huge unmet need in psychiatry to develop improvements in therapies for mental health conditions. The majority of clinical trials for new treatments fail in part due to limitations in forward and reverse translational approaches (23). Discrepancies between how behavioural constructs - in particular within neurocognitive domains - are assessed in animals and humans are a key factor in this problem.

The advent of touchscreen tasks for assessing cognitive domains in rodents (24, 25) is a significant advance for translational research. Commercially available touchscreen systems provide an opportunity to evaluate a range of cognitive constructs (e.g. learning and memory, executive function, reward learning, impulsivity and working memory) using tasks very similar/identical to those used to assess cognition in humans (e.g. CANTAB) (25–28), thereby providing superior face validity and translational potential compared to previous methods. As in human studies, animals are presented with visual stimuli (that can be easily manipulated) on a computer monitor before selecting a response. In the case of rodents this is achieved through a nose poke approach to an infra-red ‘touchscreen’ assembly.

By comparison with automated cognitive tasks in a five-hole operant chamber, touchscreen tasks offer similar advantages in that the automation enables high throughput of experiments under rigorously controlled conditions, measurement of several behaviours concurrently, the ability to test numerous subjects simultaneously, use of within-subject designs, minimisation of confounds related to animal handling, and less experimenter data analysis bias through computerised data collection. One limitation common to many operant tasks is that confounds such as motoric impairment, basic learning mechanisms and motivational factors can impact on task performance. These however can often be addressed by scrutinising variables such as trial omissions, or reaction times to collect rewards or produce a response.

Touchscreen tasks require animals to evaluate a visual stimulus on the touchscreen and approach the infrared screen to register a response without necessarily touching it (25, 27). This feature is considered to facilitate training and allows assessment of animals that may have motor impairments. However, a potential disadvantage, as compared to operant tasks that require nose-pokes to elicit a response, is that touchscreen tasks may not sustain behaviours relevant to motivation. These include paradigms such as progressive ratio (PR) for assessing motivation, and effort-related choice (ERC) related to decision-making. Importantly, Heath et al. (29) demonstrated that mice were indeed able to sustain vigorous repetitive responding in PR and ERC paradigms using the touchscreen, confirming that motivation and reward-related decision making are quantifiable in a touchscreen task. Moreover, responses to amphetamine and dopamine-receptor antagonists showed similar profiles to those found in lever and nose-poke operant versions of these tasks, suggesting that touchscreen tasks are suitable for assessing motivational factors and decision making.

Touchscreen tasks offer an advantage in that appetitive (reward) learning approaches are typically adopted (using reinforcers such as strawberry milkshake/food pellets) rather than aversive learning stimuli such as bitter-tasting food or mild electric shock. Stress is known to modulate cognitive performance, including that in IGT/rGT (30–32), so minimising any potential stressful confound is important. By utilising positive (appetitive) reinforcement and minimally stressful negative reinforcement (time-out), combined with minimal experimenter exposure through automation, touchscreen-based tasks represent an attractive strategy (33). Nevertheless, mice could be trained to obtain a large milkshake reward associated with varying probability of footstock, or a small amount of reward with no punishment in a risky decision-making task (34). However, the incorporation of aversive stimuli does have the potential to induce unwanted stress that could impact on endpoints of rGT paradigms.

The most obvious translational advantage of touchscreen-based paradigms, as compared to other operant paradigms, is that they maximise the similarity with the procedures and equipment used clinically. While the neural circuitry recruited between presentation of the visual stimulus and the ultimate selection of response and pressure on the screen in humans may not be completely characterised, it clearly makes sense to minimise any differences in procedure between species. The touchscreen approach further benefits from efficient standardisation and automation, flexibility in terms of applicability to other cognitive tasks, and minimal motor demands compared to other forms of apparatus (26, 27).

The application of touchscreen tasks in mice and rats is powerful for dissecting the impact of genetic variants upon neurocognitive behaviours, and also permits electrophysiological recordings and optogenetic manipulations to investigate the neurobiological mechanisms underpinning specific behaviours (35–38). Importantly, the ability to use similar types of visual stimuli, responses and reinforcers, using identical apparatus for different cognitive tasks, enables insight into the specific cognitive constructs important in an experimental model or manipulation. Whilst touchscreen tasks have been widely used to explore neurochemical mechanisms and neural systems important in a range of cognitive behaviours (25–27, 39–43), few studies have utilised rGT protocols.

## Genotype-phenotype relationships in the rodent gambling task

The ability of the rGT to probe multiple aspects of cognitive function (see next section) allows the role of specific genes in these aspects of cognitive function to be assessed with great sensitivity. A transcriptomic study has explored the possibility that genomic differences may underlie cautious versus risk-seeking behaviours in the touchscreen version of the task (44). A direct link with glutamatergic synapse function has been proposed, since a peptide mimetic of the actin-regulatory protein radixin, injected into the rat nucleus accumbens, altered dendritic spine morphology and reduced rGT performance in risk-averse rats (38). This gene-specific approach has proved especially informative in relation to genes associated with schizophrenia risk, and we return to this in more detail below.

## Capturing elements of gambling-related decision making in touchscreen tasks

Decision making in the Iowa gambling task integrates a wide range of neurobehavioural and neurocognitive processes. These include reward processing (to evaluate risk-reward ratio), attention (constantly to monitor information), cognitive flexibility (to account for various outcomes), inhibitory control (to refrain from choosing immediate high reward option - impulsivity), and compulsivity (persistence/perseverative responses that do not relate to achieving the overall goal).

It has long been argued that substance-use-disorder (SUD) involves a shift from being more novelty-driven and impulsive to being more habit-driven and compulsive (45, 46). Given that DSM-5 has categorised gambling disorder in the same section as ‘Substance related and Addictive disorders’ it is important to assess these behaviours in experimental models using the same task, in order to understand the neurobiological mechanisms underpinning risk and protective factors for gambling and to inform new treatments.

The touchscreen platform provides an ideal opportunity to measure a range of cognitive behaviours concurrently and to dissect the precise neural mechanisms involved in decision making in the rGT. Importantly, studies are beginning to unravel the neural mechanisms of how genetic, neurodevelopmental and environmental factors impact on different behavioural and cognitive processes in the rGT, together with the impact of drugs on performance. Here we summarise some of the approaches used to investigate these factors, along with modifications of the basic rGT that allow additional behaviours relevant to gambling to be quantified.

## Environmental and trait factors

Kim et al. (11) were the first to investigate the interaction of environmental factors with trait on decision making in a rat rGT touchscreen task using the method previously used for operant ‘nose poking’ chambers (17). Animals were trained (housed in pairs or isolation) to detect 4 choices differing in the probability and magnitude of reward (food pellet) and punishment (time-out). Once trained, they were assessed for risk-averse or risk preference behaviours under free choice conditions and their response to cocaine evaluated. Interestingly, rats could be divided into risk-averse and risk-seeking groups according to their preference for advantageous or disadvantageous choices in the free choice stage, indicating trait differences. Furthermore, pre-existing trait towards risk and the environment (housing conditions) interacted to affect decision making, and cocaine appeared to heighten this process.

## Age and impulsivity

Cho et al. (47) focussed on the impact of age on impulsivity measures in the rGT. Impulsivity can be broadly divided into ‘impulsive action’ and ‘impulsive choice’ (48). Impulsive action relates to premature responding through failure to inhibit an inappropriate response, whereas impulsive choice relates to impulsive decision making (choosing immediate rewards over more beneficial long-term rewards). Importantly, they found that rats exposed to the task early in life (late adolescents/young adults) showed increased impulsive action compared to those exposed as mature adults. By contrast, rats exposed to the task as mature adults showed an increase in impulsive choice after cocaine administration, which was only apparent in a sub-group of rats pre-categorised as ‘high impulsive action and risk averse’. These data highlight that the neurodevelopment period in which rats are exposed to the task differentially impacts on these two aspects of impulsivity, and that cocaine administration and/or stressors may be necessary to reveal differences. The neurobiological mechanisms underpinning these impulsive processes in neurodevelopment remain to be established, as well as whether they translate to human adolescence and vulnerability to gambling disorders.

## Loss chasing behaviours

One of the features of human pathological gambling is loss of control and the emergence of loss-chasing behaviour. Loss chasing is the drive to continue gambling despite successive and accumulating losses. It has been argued that different mechanisms mediate the commencement of gambling choices and their persistence, and that the neural systems recruited in the habitual nature of gambling differ from those involved in the goal-directed actions of loss chasing. To explore this, Humby et al. (7) developed a novel touchscreen task to assess gambling and loss-chasing performance under different win/loss probabilities in mice, and then assessed the impact of a 5-HT2C receptor antagonist and a 5-HT1A receptor agonist. The translational validity of the task was demonstrated as mice showed the expected patterns of behaviour when the odds for winning were altered, resembling behaviours seen in humans. Notably, antagonism of 5-HT2C receptors with SB242084 decreased the likelihood to initially gamble but did not affect loss chasing behaviour. By contrast the 5-HT1A receptor agonist 8-OH-DPAT did not affect initiation of gambling, but increased gambling choices once started. These findings support the involvement of distinct 5-HT receptors in mediating discrete components of gambling behaviours and provide the basis for further studies to explore the neurobiological mechanisms involved.

## Neural circuitry and neurotransmitters underlying IGT performance

There is good cross-species correspondence in the brain circuitry involved in IGT performance. The core circuitry recruited to perform the task in rodents includes prefrontal cortex (PFC), OFC, amygdala and striatum/accumbens (5, 19, 49). Lateral OFC is believed to be involved in the integration of historical and recent choice outcomes (50).

Serotonergic pathways contribute to various aspects of cognitive function, including attentional process, executive function and impulse control, in a distributed neural network prominently including PFC and OFC (51). They are hence likely to contribute to risk/reward processing. As noted previously, antagonism of 5-HT2C receptors in mice seemed to reduce inclination to gamble, without affecting loss-chasing, but stimulation of 5-HT1A receptors (0.03mg/kg 8-OH-DPAT) tended to increase loss-chasing (7). This may be related to results in the 5-hole operant box version of the task where 8-OH-DPAT (0.3 mg/kg) in rats increased selection of the two least advantageous options (17). The 5HT2A/C agonist LSD also failed to modify option selection in mice (8). There is also little effect of LSD in a different gambling task, without any learning component, in humans (52).

Dopaminergic pathways, both mesocortical (to PFC/OFC) and mesolimbic (to accumbens), are important for various aspects of cognitive function, including executive processes (51). Clinical studies support a role for dopaminergic pathways in IGT performance. IGT performance is impaired in early-stage Parkinson’s disease (53–55), and with cocaine exposure (56, 57), yet no overt impairment is observed after amphetamine administration (58). In rodents, the results for indirectly-acting dopamine agonists with the touchscreen rGT largely parallel those obtained using the 5 hole operant box. Acute administration of cocaine (15mg/kg) had little overall effect on choice selection in rats (11, 47). Rats receiving a cocaine challenge (15mg/kg), having been withdrawn for 2 weeks after a week of repeated cocaine exposure, showed decreased selection of the optimal choice, and increased selection of the second-worst but not the worst option (11). However, a low dose of amphetamine (1.5mg/kg) in mice slightly increased selection of the 2^nd^ best option, without affecting premature responses (10). A marginally higher dose (2mg/kg), also in mice, reportedly had no significant effect on choice selection, but with a tendency towards increased selection of the 2^nd^ best option, while decreasing premature responses and increasing omission rates (8). In rats, amphetamine (0.3-1.0 mg/kg) also increased selection of the 2^nd^ best option at the expense of the best option, while additionally increasing premature responding (17, 59–61). The same effect is seen with 2mg/kg amphetamine in mice (62), although here it was omissions that increased rather than premature responses.

The reason why amphetamine leads to slightly less advantageous responding, and in particular elevated responses at the 2^nd^ best option, are unclear. Amphetamine does tend to increase impulsivity and slightly compromise attentional task performance in rodents (63). It may be that there is a reduction in the acuity of risk/reward assessment, but combined with an increased awareness of harm avoidance, leading to increased selection of the 2^nd^ best choice, but not the least advantageous choices associated with greater levels of punishment.

Localised overexpression of D1 dopamine receptors in rat PFC increased selection of disadvantageous choices in the touchscreen rGT, and was interpreted as increased risk-taking (64). An agonist at D3 dopamine receptors increased selection of risky options in the 5 hole box rGT (65).

Pathological gambling behaviour is a particular problem associated with the use of directly acting dopaminergic agonists to treat Parkinson’s disease (66, 67). Pramipexole, an example of drugs of this class, increases risk-taking behaviour in the IGT in control subjects and in people with early Parkinson’s disease (68). Similar effects are observed in people with bipolar disorder (69). Equally, pramipexole is recently reported to increase selection of the 2^nd^ worst option in control mice, and to increase selection of the least advantageous choice in mice with lesions of the dopamine system, using a touchscreen rGT (70). Hence there seems to be strong translational relevance of the rGT with dopaminergic agonists.

Modafinil, which facilitates dopaminergic and noradrenergic transmission via uptake transporter inhibition, along with other actions as well, reduced loss-chasing behaviour in a simplified touchscreen gambling task (7). No effect on choice selection was detected in a 5-hole operant box version of the task (62). The selective noradrenaline uptake inhibitor atomoxetine, administered acutely, did not affect gambling behaviour, including loss-chasing, but reduced premature responding (60, 61). Noradrenaline is known to play an important role in attention and executive processes (51, 71, 72). Interestingly, the alpha2 adrenoceptor agonist clonidine, while not affecting IGT performance in control subjects, produced a fairly clear improvement in heroin-users (73). A long-standing literature suggests functional links between alpha2 receptors and opioid receptors (74).

The widespread role of glutamatergic signalling in cognitive processes, in particular by NMDA receptors, would suggest that interfering with NMDA receptor signalling might impair performance on the IGT/rGT. Interestingly, just as with amphetamine, an NMDA receptor antagonist increases selection of the 2^nd^ best option at the expense of the most favourable option in the 5-hole operant chamber rGT, while also dramatically increasing premature responses (75).

## Schizophrenia, depression and bipolar disorder

These psychiatric conditions arise due to a combination of genetic and non-genetic factors. In the case of schizophrenia, genetic advances have to some extent confirmed pre-existing ideas, based on drug effects, that there might be some fundamental alterations in the activity of dopaminergic and glutamatergic pathways (76, 77). These alterations are thought particularly to affect the neural circuitry centred on thalamic connections to PFC, OFC and hippocampus (78–80). Neurotransmitter disturbances in depression and bipolar disorder are less thoroughly investigated, but PFC/OFC dysfunction is also a core component of these conditions (81–84).

Compared to control subjects, people with schizophrenia, depression or bipolar disorder all show similarly altered levels of performance in the IGT (85–88), with reduced selection of the best choice and increased selection of the second worst choice (89, 90) or worst choice (91–93). The deficits observed in people with schizophrenia are particularly robust (88, 93–104). In depressed people, symptom severity correlates with harm avoidance, that is a tendency to avoid the least advantageous options (105).

A modification of the IGT that incorporates a contingency-shift element has proved especially informative for revealing altered cognitive risk/reward processes (103). In this modification, the reward/punishment contingencies associated with each choice option are modified during the course of the test session, this providing an indication of ability to adjust to differing risk/reward assessments and adapt behaviour accordingly. People with schizophrenia who were able to achieve levels of performance similar to controls in the baseline test then experienced difficulty in adjusting their responses when the contingences of 2 of the choices were switched. This could be interpreted as “harm avoidance”, manifest as a reluctance to select choices previously linked to unfavourable outcomes (103). There is some evidence that people with schizophrenia are particularly sensitive to the “worst” option, despite their impaired overall IGT performance (106). Anxiety, which is often present in schizophrenia (107, 108), is thought to impair IGT and rGT performance (5, 109), and can lead to avoidance of the worst IGT options (110). People prone to hallucinations/delusions were reported to perform at normal levels in the basic test, but to show impaired performance after the contingency shift (111), further suggesting that the manipulation increases sensitivity to detect impairment. People with depression are also impaired during this contingency-shift stage (90), showing an unwillingness to shift to the “previously bad, now good” choices. People with bipolar disorder taking the D2/D3 agonist pramipexole become more sensitive to gains than to losses (69).

## Genetic manipulations relevant to schizophrenia and other neuropsychiatric conditions

**Figure 1:**
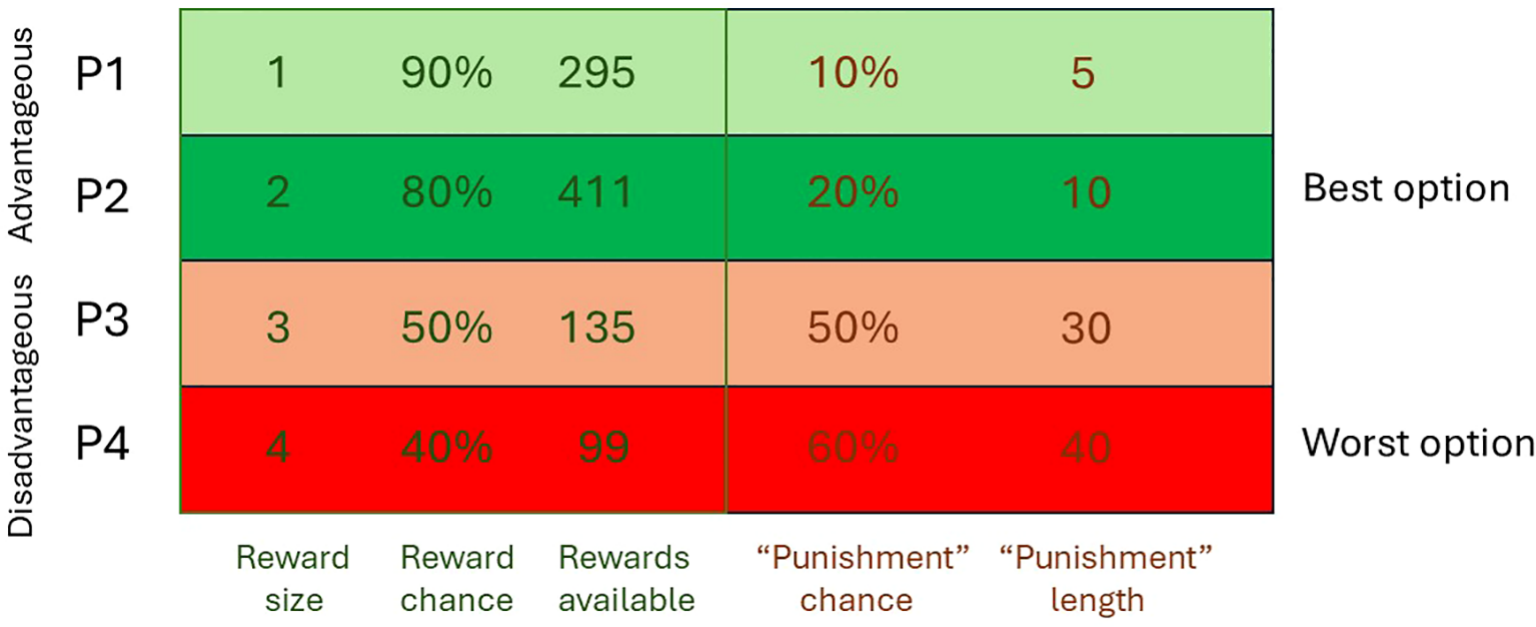
Task design and risk/reward contingencies as used by Thomson et al. (9).

We have adopted the touchscreen task to assess the impact of genetic manipulations relevant to schizophrenia upon mouse gambling performance, using four reward punishment contingencies based upon Zeeb et al. (17). The design of the task and the parameters used are summarised in **Figure 1**.

GPR88 is an orphan G protein couple receptor enriched in striatal GABAergic medium spiny neurons, but also present in PFC (112). Based upon this location, GPR88 receptors are strategically placed to modulate the function of several cortico-striato-thalamo-cortical loops, suggesting potential utility as a target for treating schizophrenia and other neuropsychiatric conditions. Indeed, there is an interaction between Gpr88 and dopaminergic function (113–115), and we and others have reported impairments in mice lacking *Gpr88* in cognitive domains involving PFC/OFC circuitry (9, 116).

*Gpr88* KO mice show a perturbance of reward processing, selecting more risky choices (P4) at the expense of more advantageous, lower risk options (P1). At the same time, mice showed increased premature responding indicating motor impulsivity. These findings suggest a hyperdopaminergic phenotype, and together with performance in other tasks suggest that GPR88 KO mice are a useful model to evaluate novel targets for a range of cortico-striato-thalamo-cortical-mediated behaviours relevant to schizophrenia and other neuropsychiatric conditions (9). Hence these mice provide an opportunity to evaluate compounds and investigate neurobiological mechanisms from a transdiagnostic perspective.

We noted the particular difficulty that people with schizophrenia, or those prone to delusions and hallucinations, experience in the contingency-shifting modification of the IGT (103, 111). Common sequence variants in the *MAP2K7* gene roughly double risk of schizophrenia (117). This is a large effect size for a common variant, making the gene of great interest for functional investigation in rodents. Mice heterozygous for deletion of the *Map2k7* gene showed altered prepulse inhibition of the startle reflex, hyperlocomotion, and increased levels of omissions in a touchscreen attentional test, alongside reduced metabolic activity in the prefrontal cortex (39, 118). This is a pattern of phenotypes that closely relates to schizophrenia. There is also evidence for altered dopaminergic function in this strain of mice (118). However, when tested in the standard rGT, the mice performed at equivalent levels to control mice (10). We therefore incorporated a contingency-shifting component. When the two intermediate contingencies were switched, the *Map2k7* hemizygous mice adapted as rapidly as the wild-type mice, showing no evidence for perseveration for the previous learned responses. However, when the best and worst options were switched, we found that the *Map2k7* hemizygous mice were dramatically impaired, essentially being unable to complete the shift back to a more optimal choice selection, despite large numbers of retraining sessions (10). They continued to avoid the option that had previously been the worst choice. We hypothesise that the *Map2k7* hemizygous mice are showing a form of harm avoidance behaviour, and that full function of the *Map2k7* gene is necessary for overcoming this anxiety-related contribution to cognitive flexibility. Indeed, there is some evidence linking the *MAP2K7* gene to panic disorder (119).

## Conclusions

In summary touchscreen tasks are showing great promise as translational tools to dissect neurobiological mechanisms in the rGT. Since maladaptive decision making occurs in many psychiatric conditions, examining genetic, neurodevelopmental and environmental risk factors for these conditions in the rGT could provide greater insight into how these factors may cross current diagnostic boundaries and offer transdiagnostic opportunities to evaluate novel treatments. These studies also pave the way to dissect the specific neural circuits and neurotransmitter mechanisms underpinning distinct behaviours though the concurrent use of optogenetic approaches and *in vivo* electrophysiological recordings during evaluation of behaviours.
